# Embodied Dyadic Interaction Increases Complexity of Neural Dynamics: A Minimal Agent-Based Simulation Model

**DOI:** 10.3389/fpsyg.2019.00540

**Published:** 2019-03-21

**Authors:** Madhavun Candadai, Matt Setzler, Eduardo J. Izquierdo, Tom Froese

**Affiliations:** ^1^Program in Cognitive Science, Indiana University, Bloomington, IN, United States; ^2^School of Informatics, Computing, and Engineering, Indiana University, Bloomington, IN, United States; ^3^Institute for Applied Mathematics and Systems Research (IIMAS), National Autonomous University of Mexico (UNAM), Mexico City, Mexico; ^4^Center for the Sciences of Complexity (C3), UNAM, Mexico City, Mexico

**Keywords:** social interaction, agent-based models, artificial neural networks, evolutionary robotics, embodied cognition

## Abstract

The concept of social interaction is at the core of embodied and enactive approaches to social cognitive processes, yet scientifically it remains poorly understood. Traditionally, cognitive science had relegated all behavior to being the end result of internal neural activity. However, the role of feedback from the interactions between agent and their environment has become increasingly important to understanding behavior. We focus on the role that social interaction plays in the behavioral and neural activity of the individuals taking part in it. Is social interaction merely a source of complex inputs to the individual, or can social interaction increase the individuals' own complexity? Here we provide a proof of concept of the latter possibility by artificially evolving pairs of simulated mobile robots to increase their neural complexity, which consistently gave rise to strategies that take advantage of their capacity for interaction. We found that during social interaction, the neural controllers exhibited dynamics of higher-dimensionality than were possible in social isolation. Moreover, by testing evolved strategies against unresponsive ghost partners, we demonstrated that under some conditions this effect was dependent on mutually responsive co-regulation, rather than on the mere presence of another agent's behavior as such. Our findings provide an illustration of how social interaction can augment the internal degrees of freedom of individuals who are actively engaged in participation.

## 1. Introduction

Social interaction has become a hot topic in cognitive science. Not too long ago a radical individualism about collective phenomena was the only game in town, leading respected philosophers to conclude that ultimately the basis of our mental life does not depend on others at all, such that it would make no difference if others were just a hallucination of a “brain in a vat” (Searle, 1990). Nowadays there is a growing consensus that this pessimistic view is inadequate, and that social interaction can make a difference to the mental and behavioral activity of individuals (Froese, 2018). For instance, evidence from neuroimaging, psychophysiological studies, and related fields has revealed that the mechanisms of social cognition are different when we are in real-time interaction with others compared to when we are passive spectators (Schilbach et al., 2013).

Nevertheless, the extent and nature of the influence of social interaction on an individual is still contentious. Most researchers adopt a moderate individualism in which interaction with others can make a difference but only externally so, for example by serving as a source of additional information, by having a causal influence, or by providing an opportunity for adopting a more socially oriented mode of cognition (Gallotti and Frith, 2013). Other researchers adopt an enactive approach that questions the validity of this restriction, proposing instead that the interaction in itself can play a role in realizing an individual cognition, thereby transforming and augmenting the individual capacities (De Jaegher et al., 2010). On this latter view, social interaction could allow an individual to overcome the limitations of their individual capacities by incorporating the complex dynamics of the interaction process into the basis of their internal activity.

Agent-based modeling offers a suitable framework with which to start investigating this possibility in a systematic manner. In particular, by simulating pairs of mobile agents in highly simplified scenarios it becomes possible to systematically assess the relationship between individual complexity and social interaction (Froese et al., 2013b). For instance, in previous work one of us provided a proof of concept that evolving two agents to locate each other in an open-ended arena via acoustic coupling can result in activity in their neural controllers, which in principle would have been too complex for them to generate in isolation (Froese et al., 2013a). Here, we show that this is not an isolated finding: directly evolving pairs of agents to increase the complexity of their neural activity consistently results in behavioral strategies involving mutually coordinated interaction between them. Moreover, we show that there is a crucial difference between forms of interaction in which the agents behaviors are interdependent compared to independent from each other: neural complexity achieved during mutually coordinated interaction tends to be even higher than what can be achieved during one-way coordinated interaction.

## 2. Materials and Methods

**Figure 1:**
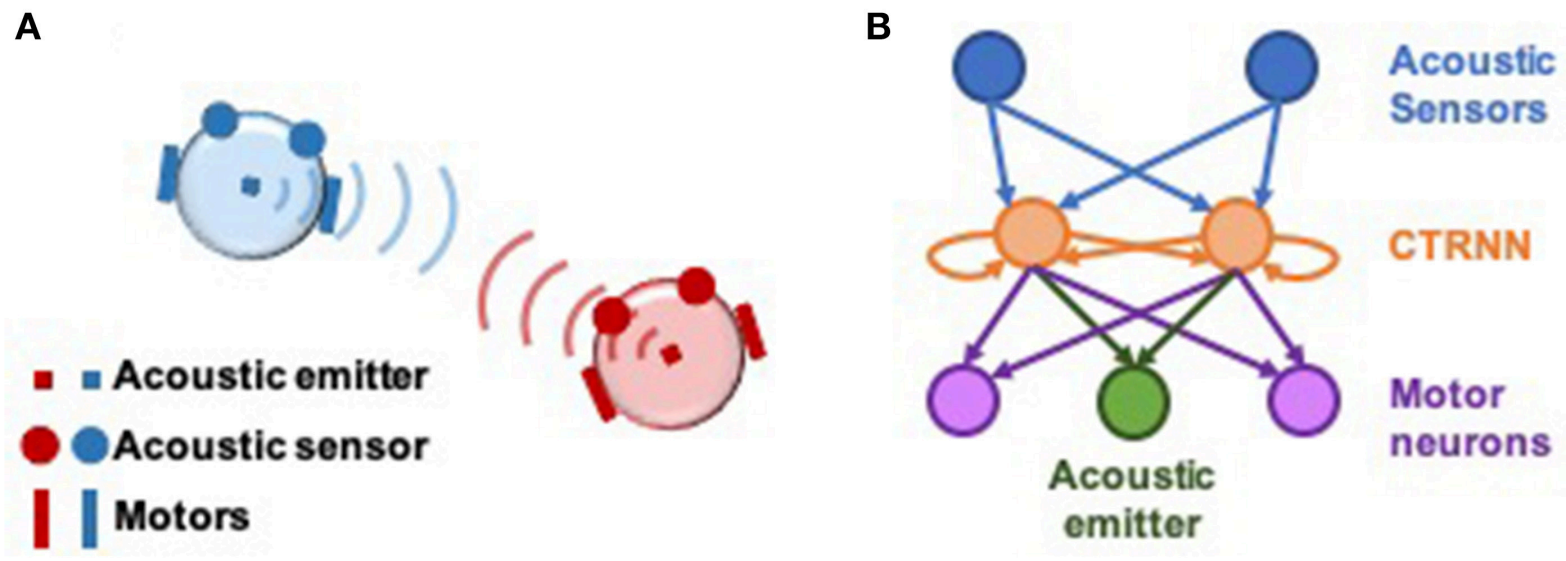
Setup of computational model and neural network architecture. **(A)** Illustration of socially interacting agents. Two agents, each consisting of an acoustic emitter that they are able to modulate, a pair of acoustic sensors to sense the other agent, and two motors to move in a 2-dimensional environment. The ability to modulate their own signal combined with their ability to listen to their counterpart, enables interaction in this model. Agents cannot sense themselves. **(B)** Neural architecture of the agents. The two acoustic sensors feed into a 2-neuron fully-connected continuous-time recurrent neural network (CTRNN) circuit which in turn feed into the two motors and the acoustic emitter. The movement of the agent is result of the net activation of the left and right motor neurons.

Experiments were conducted on pairs of simulated agents that interacted with one another in an empty 2-dimensional environment. Each agent emitted an acoustic signal, which could be sensed by the other via two sensors positioned at the perimeter of their circular bodies (Di Paolo, 2000). The strength of the emitted signal faded linearly with distance, and sensors were positioned to be 90° apart from one another (Figure 1). Thus, agents can gather information about their relative distance and orientation to one another. Neural controllers were modeled as dynamical recurrent neural networks (Beer, 1995). Sensory input filtered through sensory neurons into an inner layer of two interconnected neurons, whose activity modulated the power of the emitted acoustic signal and controlled motor neurons that propelled the agent around its environment.

Parameters of the neural controllers, such as weights and signs of the connections, biases, and time-constants, were optimized using an evolutionary algorithm. Each evolutionary run was initialized with a random population of 96 solutions, that was evolved over 500 generations. Hundred such runs were executed and the best solution in the population from each run was collected to be analyzed. In order to evaluate the fitness of the individuals, we computed the entropy of the time series of neural activity taken from simulated trials. This measure allowed us to operationalize the complexity of internal neural dynamics exhibited by each agent in various interaction conditions. In particular, neural entropy was measured for each agent in trials where they were evolved and interacted in pairs (*interaction entropy*), as well as control conditions where agents were placed in the environment by themselves (*isolation entropy*). Our decision to use neural entropy as an index of internal complexity was motivated by its interpretability and computational tractability, as well as a range of previous studies that have associated elevated levels of neural entropy with improved cognitive performance, including therapeutic benefits (Carhart-Harris et al., 2014), increased levels of consciousness (Schartner et al., 2017), and improved generalization in motor learning tasks (Dotov and Froese, 2018). Please refer to the Supplementary Material for more details on the parameters of the evolutionary optimization methodology adopted.

## 3. Results

### 3.1. Interaction Enhances Internal Complexity Beyond What Is Possible Alone

**Figure 2:**
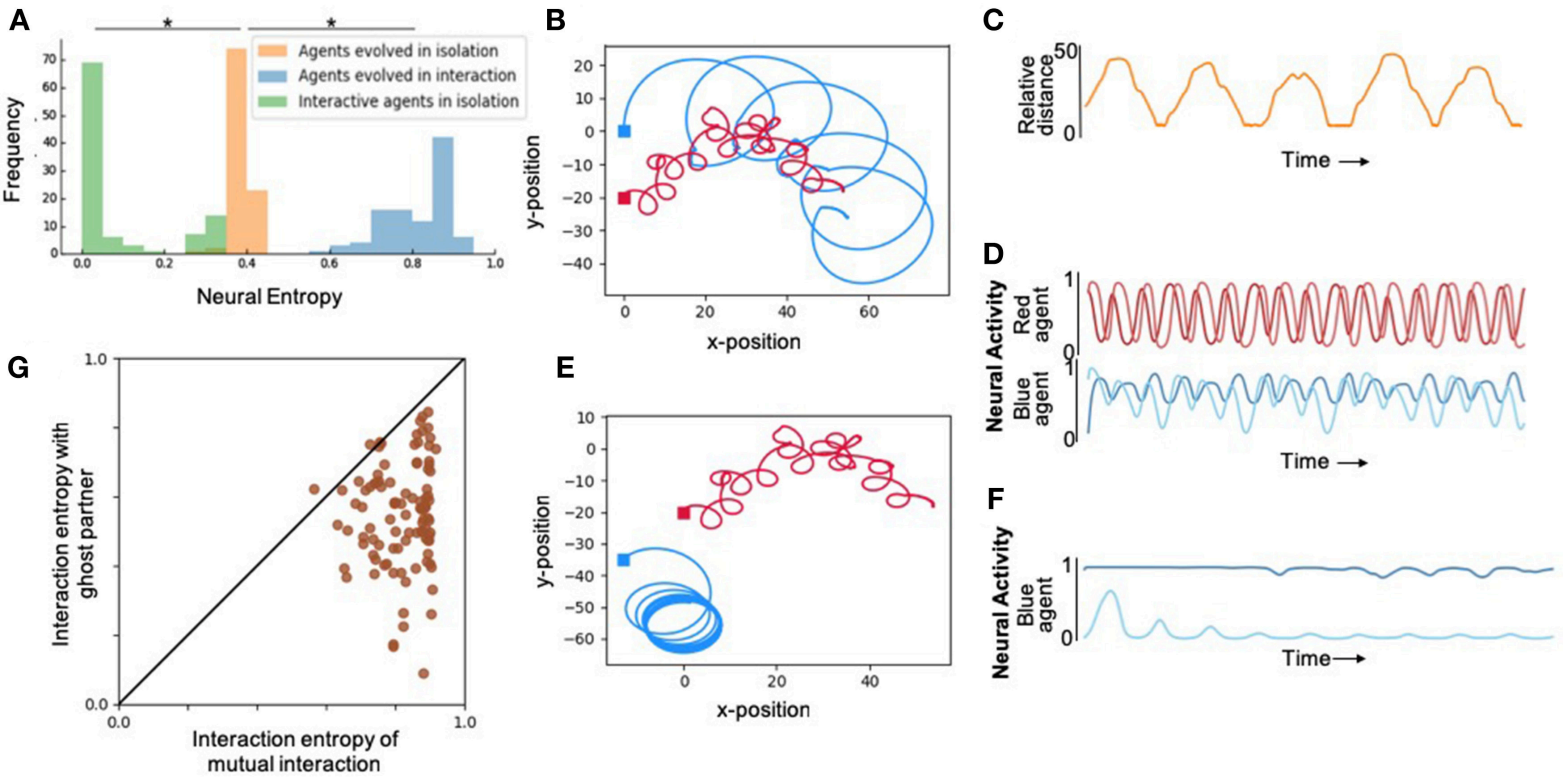
Results depicting effect of social interaction on neural complexity. **(A)** Fitness distributions of best agent in the population from 100 runs for each of the different levels of social interaction. Agents evolved with interaction (blue) showed highest neural complexity, however, when the same agents were evaluated in isolation (green) showed significantly lower neural complexity even compared to agents evolved in isolation (orange). *Denotes statistically significant difference with *p* < 0.005 (see Supplementary Material for details). **(B)** An illustration of the 2-dimensional behavioral pattern of two agents evolved to interact demonstrating aperiodic oscillatory patterns that cannot be achieved by the 2-neuron systems of each agent in isolation. **(C)** Relative distance over time of the two agents shown in **(B)**, also demonstrating interesting complex patterns that cannot be achieved by passive 2-neuron CTRNNs. **(D)** The neural activity of the 2 interneurons of red and blue agents shown in **(B)**, demonstrating chaotic aperiodic activity that cannot be generated by 2-dimensional CTRNNs in isolation in the absence of interaction. **(E)** The same agents as in **(B)**, but in this case the red agent plays back the recorded behavior from the trial shown in **(B)**, while the blue agent is allowed to interact with it. Significantly reduced behavioral complexity is observed under this “ghost” condition where agents are unable to mutually interact with each other. **(F)** Neural activity in interneurons of blue agent under the ghost condition, showing significantly lower complexity compared to the same agent's neural activity in the interactive mode shown in **(D)**. **(G)** Neural entropy and behavior in the presence of an active partner vs. ghost partner. All agents exhibit high values along the horizontal axis demonstrating high internal complexity in the presence of responding partners. However, as it can be seen from the spread along the vertical axis, below the diagonal, these agents lose internal complexity when their partner is a ghost. This loss tends to be more pronounced for higher levels of interaction entropy, which suggests that these higher levels are more readily achieved by interdependent rather than independent interaction.

First, in order to study the effect of interaction on internal complexity, we artificially evolved pairs of agents to maximize their interaction entropy, without explicitly specifying any desired behavior. The resulting movement and neural traces from one trial of one of the best evolved pairs of agents from 100 runs is shown in Figure 2. During interactions, agents exhibited normalized neural entropies of 0.7568 and 0.8763. Although behavioral interactions were not selected for, evolved agents exhibited a complex pattern of moving toward and away from each other in a coordinated manner (Figure 2B, Supplementary Figure 5). Qualitatively similar behaviors were observed in the rest of the evolutionary runs (Supplementary Material).

We expected that agents would evolve to make use of social interaction to enhance their internal complexity, if there was an opportunity to do so. In order to verify this prediction, we performed another set of experiments where we evolved isolated agents using the same fitness function. Comparing the neural entropy achieved by agents in 100 independent evolutionary runs in each condition revealed that internal complexity was significantly higher when agents had the ability to interact as opposed to when they existed in isolation (Figure 2A). In other words, the interaction entropy of agents evolved in social contexts is consistently larger than the isolation entropy of agents evolved in isolation.

### 3.2. Complex Interactive Behavior Does Not Require High Isolation Entropy

From the previous results, it does not directly follow that agents that show high interaction entropy would also exhibit high isolation entropy. This is an empirical question regarding the emergence of complex interactive behaviors from simple systems. In order to test this, we disabled the sensors in agents that were optimized in interactive environments and measured their neural entropy in isolation. These agents consistently showed lower levels of entropy than what they exhibited during interaction (Figure 2A). Importantly, all of these agents also showed significantly lower levels of entropy than what was typically achieved by agents evolved in isolation to maximize isolation entropy (Figure 2A). In other words, although these agents were more complex during interaction, they are not intrinsically more complex. This has implications for developmental psychology, since these results suggest that complex interactive behaviors do not require high intrinsic internal complexity, as long as infants have the capacity to take advantage of the complexity provided by interaction.

### 3.3. Agents Exhibit Higher-Dimensional Dynamics During Interaction

From a dynamical systems perspective, in isolation, these simulated mobile robot systems are two-dimensional autonomous systems (2 neuronal states) that can at most have fixed-point or limit-cycle attractors (Beer, 1995). During the course of interaction with another agent, these dynamical systems show aperiodic dynamics more complex than limit-cycles and that in principle require at least 3 dimensions (Figure 2D). In the presence of another agent, the coupled system is of higher dimensionality involving both agents and their relative environmental states. In this case, measuring the neural entropy in one agent neural activity is akin to measuring the entropy of the two-dimensional projection of a higher dimensional system. This explains the enhanced levels of internal complexity in agents that are in the presence of others through their interaction the two embodied agents can become integrated into a larger, dynamically extended system (Froese and Fuchs, 2012).

This dependence on interaction with their partner to enhance neural complexity, and hence behavioral complexity, could be from two categorically different underlying interactive modes.

1. The partner could be a source of complex stimuli that drives the agent in question to perform behaviors through complexification of neural dynamics. In this case, the other agent becomes a passive component of a complex environment that the agent in question uses to realize complex neural dynamics. We refer to this mode as *independent interaction*.
2. The two agents could be engaged in mutually interdependent interactive behaviors, thereby bootstrapping neural complexity in each other through continuous interaction via acoustic modulation and spatial navigation. In this case, the other agent is no longer passive but is an active responsive component that continuously influences and is influenced by the neural dynamics of the agent in question. This mode of interaction is henceforth referred to as *interdependent interaction*, which is a generic form of coordination.

### 3.4. Internal Complexity Is Enhanced More by Interdependent Interaction

In order to disambiguate the aforementioned two modes of independent and interdependent interaction, we measured interactive entropy in the presence of “ghost” partners. “Ghosts” were agents that were merely playing back their movements from a previous trial, without being responsive to the “live” agent whose neural entropy is being measured. The “ghost” condition preserves complexity of the signal that the “live” agent experiences, nevertheless, it does not present any opportunity for interdependent interaction or coordination. Under the ghost condition, live agents suffered a loss in internal complexity in most cases. This demonstrates that their neural complexities were enhanced by active interdependent interaction with the other agent, and not just because of the presence of complex driving signals (Figure 2F).

The same pair of agents described in Figure 2B were examined again in Figure 2E. This time, however, one of the agents was made into a “ghost” (same movement as before, but unresponsive to environmental feedback). As a result from this change, the live agent's behavior becomes starkly different and its entropy drops to 0.4712. This shows that the agents did not simply rely on the complex sensory stimuli from the behavior of the other agent. Instead, the two agents were mutually interacting: they were coordinating their movements and were thereby enhancing each other's neural and behavioral complexity in a complementary manner. More generally, we found a statistically significant correlation between increasing internal complexity and interdependent interaction, and that this form of interaction tended to be more ordered, as would be expected from social coordination (see Supplementary Material for details).

## 4. Discussion

From a complex systems perspective we expected that placing embodied agents in an interactive context would transform their neural and behavioral dynamics, and that certain forms of interaction would lead to an increase in their complexity. Our modeling results confirmed this expectation by providing a proof of concept that the behavior of embodied agents in real-time dyadic interaction cannot be fully understood from studying their brains in isolation, nor even in the context of non-responsive social stimuli.

In our simulation model an agent's neural complexity could increase beyond its individual degrees of freedom when the agent is interacting with a complex environment, and especially so when it is coordinating its behavior with another responsive agent. Our analysis revealed that this increase is not just a matter of activating latent internal complexity: interaction allows an agent's neural activity to increase its complexity to such an extent that in principle it would be impossible for that activity to be generated in isolation. This finding suggests that the enactive approach to social cognition is on the right track: the dynamical basis of an agent's behavior during real-time interaction with another agent becomes the whole brain-body-environment-body-brain system (Froese et al., 2013b), of which each agent brain is just one important component (Gallagher et al., 2013) whose neural activity becomes a projection of the overarching interaction process. Future modeling work could analyze in more detail how this interactive expansion of individual complexity is dynamically realized, for example by analyzing the transformation of the state space of the overarching brain-body-environment-body-brain system as it goes from an uncoupled to a coupled mode. It also remains to be seen to what extent this increase in individual complexity scales with the number of individuals that are interacting.

Another avenue for future investigation is to verify these modeling findings in the context of actual human social interaction. The so-called “second-person” approach to social cognitive neuroscience has already revealed that the brain is activated differently when participants are engaged in real-time social interaction when compared to passive observer scenarios (Schilbach et al., 2013). The complex systems perspective adopted by the enactive approach could help to provide an explanation for this observed difference. More specifically, it would be interesting to verify our finding that an agent's neural activity tends to be transformed more substantially in scenarios involving interdependent compared to independent forms of interaction between agents. Importantly, our results reveal that interpersonal behavioral synchrony in itself is not sufficient to distinguish between interdependent and independent forms of interaction. Accordingly, future experimental work could compare neural activity in a task requiring real-time coordination with neural activity in a non-responsive “playback” control condition, for instance by employing the human dynamic clamp paradigm (Dumas et al., 2014).

## Data Availability

The datasets generated for this study are available on request to the corresponding author.

## Author Contributions

TF conceived of the presented idea. All authors designed the experiments. MC, MS, and EI developed the code. MC carried out the experiments and analysis of the data. All authors discussed the results. MC was the main contributor to the Methods and Results of the manuscript with input from all authors. TF was the main contributor to the Introduction and Discussion of the manuscript with input from all authors. All authors discussed and contributed to the writing of the final manuscript.

### Conflict of Interest Statement

The authors declare that the research was conducted in the absence of any commercial or financial relationships that could be construed as a potential conflict of interest.
