# Supplementary material for: Embodied Dyadic Interaction Increases Complexity of Neural Dynamics: A Minimal Agent-Based Simulation Model
Source: Front Psychol. 2019 Mar 21;10:540. doi: 10.3389/fpsyg.2019.00540 (PMC6437094; doi:10.3389/fpsyg.2019.00540)
Supplement: Supplementary file 1 [file Data_Sheet_1.PDF]

Supplementary Material: Embodied dyadic interaction increases complexity of neural dynamics: A minimal agent-based simulation model

This supplementary material document contains a detailed materials and methods section, additional results, additional figures that illustrate behaviors of agents from different runs, and results for statistical tests of significance that were conducted.

1 MATERIALS AND METHODS

This section outlines the implementation details to recreate the simulated agents, and all experiments and analyses. We first describe the agent, their neural circuitry and their principles of movement and acoustic signaling, followed by an explanation of the evolutionary stochastic search algorithm used to optimize these agents, and then a description of the analyses is provided.

1.1 Agent design

The design of the simulated robots has been primarily inspired by Di Paolo's acoustically coupled agents (Di Paolo, 2000). The simulated agents were circular with a radius, R, of 4 units and were equipped with two acoustic sensors and an emitter. The two sensors were located symmetrically at an angle of 45 degrees to the central axis. An acoustic emitter was located at the geometric center of the body, and hence equidistant to an agent's own sensor making them essentially deaf to their own signal. The emitted signal experiences linear loss in strength with distance. The strength will be maximum and equal to that of the emitted strength at a distance equal to the 2R between the center of the agents and linearly drops off with increasing distance. The sensors that pick up this signal hence provide an estimate of the distance of the source and the differential sensory stimuli in the two sensors give an estimate of the direction of the source relative to the sensing agent (Fig. 1A).

In addition to attenuation due to distance, the sensing agent also experiences attenuation when the signal passes through its own body – a self-shadowing mechanism that is a natural consequence of embodied agents. This attenuation is computed as a scaling factor over the sensory inputs which ranges from 0.1 to 1, depending on the proportion of the body's diameter that the signal travelled through i.e. the sensory signal is scaled by 0.1 if the sensors are diametrically opposite from the source and scaled by 1 if the agents is facing the source. This is computed as follows - for a distance D between the two agents, and a distance of D_{sen} between the source and the sensor, the shielded distance that the signal travels through the body, D_{sh}, is given by

$$\begin{aligned} D_{sh} &= D_{sen}(1 - A) \;\; 0 \leq A \leq 1 \\ A &= (D^2 - R^2)/D_{sen}^2 \end{aligned} \tag{S1}$$

where when $A \geq 1$, there is an unobstructed line between the source and the sensor. The sensory input for a sensor is first calculated based on the distance between the sensor and the source, and it is then multiplied by a scaling factor between 0 and 0.1 by linearly mapping D_{sh} between 0 and 2R respectively. This process was then repeated for the other sensor.

Collisions were modeled as point elastic, meaning that upon colliding, the agents experienced no change in their angular velocity (no friction between bodies) and momentum of the entire system was conserved by having zero net effect on their velocity vectors. This was performed by simply exchanging the velocity vectors of the agents thereby causing them to bounce off of each other without losing any energy in the process.

The internal neural circuitry is made up of three layers – sensory layer, interneuron layer and the motor control layer (Fig. 1B). The sensory neurons can be thought of input neurons with a sigmoidal activation function. Their output is given by

$$o_s = g_s \sigma(I_s + \theta_s) \tag{S2}$$

where $\sigma(x) = 1/(1 + e^{-x})$ is the sigmoidal activation function, g_s is the sensory gain that is kept the same for both sensors, and θ_s is the bias that is also same for both sensors.

The interneuron layer is a continuous-time recurrent neural network (CTRNN) that is fully recurrently connected. This corresponds to a two-dimensional dynamical system with the activity in each neuron governed by the following state equation

$$\tau_i \frac{dy_i}{dt} = -y_i + \sum_{j=1}^{N} w_{ij} \sigma(y_j + \theta_j) + \sum_{s=1}^{2} w_{is} o_s \tag{S3}$$

where dy_i/dt refers to the rate of change of internal state, y_i of neuron i based on a time constant τ_i. This rate of change depends on three values – the current state, the weighted sum of outputs from all N neurons in the network, and the total external input. The input from other neurons is calculated by weighting their output with weights from neuron j to i specified by w_{ij}. The output of each neuron based on its internal state is given by $\sigma(y_j + \theta_j)$ where θ_j refers to a bias term for that neuron. Finally, the state is also influenced by the total external input received by the neuron, in this case given by the weighted sum of the sensory input with weights w_is from sensory neuron s to interneuron i and o_s being the sensory output from two sensors.

The interneurons feed into the motor control layer, where the input to each motor neuron is a weighted sum of the outputs of the interneuron. The motor control layer contains three neurons, two corresponding to the left and right motors and one that corresponds to the acoustic signal emitter. All three of them are sigmoidal units with a gain and bias but no internal state such that the output of unit i, m_i, is given by

$$m_i = g_m \sigma(\sum_{n=1}^{N} w_{ni} * o_n + \theta_i) \tag{S4}$$

where o_n is the output of the interneuron, that are weighted by w_{ni} and θ_i is the bias term that is common across all motor units, and so is their gain g_m.

Locomotion is controlled by the effective control of the two motors, where net linear velocity is given by the average of their outputs and the angular velocity which rotates the agent and hence the direction of movement is given by their difference divided by the radius of the agent.

1.2 Measuring neural entropy

The neural activity in an agent is recorded during the course of behavior, and the neural complexity is measured as the entropy in the two-dimensional time series from the outputs of the two interneurons. Since these are outputs from a sigmoid function, they are bounded between 0 and 1. The output space is binned with 100 bins along each dimension, totaling 10000 bins in all. A 2-dimensional histogram is built from binning data points collected over all trials of the behavior and the probability of the neural activity in a bin [i,j], p_{ij}, is given by the number of points in that bin divided by the total number of points. From these probabilities, the entropy H of the neural time series is given by

$$H = \sum_{i=1}^{100} \sum_{j=1}^{100} -p_{ij} log(p_{ij}) \tag{S5}$$

The maximum possible entropy that can be achieved is when all bins are uniformly populated giving a uniform distribution over the two-dimensional histogram. The entropy computed is normalized to be in the range [0,1] by dividing by this maximum entropy which is equal to log(100*100). Thus, normalized entropy is $\hat{H} = H/log(100 * 100))$.

1.3 Evolutionary optimization

A real-valued stochastic search algorithm was used to optimize these agents to maximize their neural entropy. Each agent had 20 parameters that need to be tuned, and they were encoded as the genotype of the evolutionary search. For K agents, the genotype contained 20K values in the range [-1,1] that were scaled appropriately to construct the agent. The sensor and motor gains were scaled to be in [1,5] while their biases were scaled to be in [-3,3]. All weights were scaled to be in the range [-8,8] while the time-constants in the CTRNN were set in the range [1,2] and their biases were scaled in [-3.3]. Thus, one genotype representing one solution was mapped to construct the agent(s) to then be simulated and evaluated for performance.

One generation of evolutionary optimization involved evaluating a population of 96 solutions and generating a new population for the next generation based on their performance. Agent(s) built from each genotype were evaluated over 4 independent trials each lasting 200 simulation seconds at a step size of 0.1. Their neural activity was recorded, and entropy was computed over the 4 trials and the normalized neural entropy was assigned as their fitness.

In the interaction scenarios, the agents were always placed at a distance of 20 units from each other, but their relative angle was varied as $[0, \pi/2, \pi, 3\pi/2]$ for each trial. The fitness of each genotype in the multi-agent runs was computed as the average neural entropy of all agents that the genotype encoded. In order to promote interaction, and avoid the case where agents moved too far from each other and not be able to sense the other, a distance threshold of 100 units was set. Any trial where the agents moved further than 100 units from each other was cut-off.

Upon evaluating their fitness, an elite fraction of the top 4% solutions were kept as is, and a new population was created by creating the remainder of the solutions by mutating and crossing over these elite solutions. Mutation involved adding a zero-mean Gaussian mutation noise with variance 0.1 to the

solutions. Following that, they were crossed over with other solutions such that each parameter between a pair of solutions was swapped with a probability of 0.1. This process was repeated over 500 generations and the best solution from the population at the end of 500 generations was chosen as the representative solution from that run. 100 such independent runs were executed for each scenario discussed in the results section, and the analyses were carried out from the collection of the 100 best agents from all these runs.

There are two different scenarios under which agents are evolved - isolation and interaction. When evolved in isolation, the genotype encodes the parameters of a single agent, and the fitness of each agent is evaluated by the entropy of its neural activity during and evaluation period. On the other hand, when agents are evolved with interaction, they are evolved in pairs. In this case, each genotype encodes the parameters for two agents. The pair of agents built from the genotype can now potentially interact with each other to influence each other's neural activity. Fitness for each genotype is then evaluated as the average neural entropy of the two agents.

1.4 Measuring distance entropy

The distance between the two agents, unlike their neural entropy, is a one-dimensional time series. During the optimization trials, the distance between agents was set to a maximum limit of 100 units, beyond which the trial was cut off. In order to maximize fitness all evolved agents optimized their behavior to stay within this bound. This range of distance ([0,100]) was binned into 100 bins, which allowed the creation of one-dimensional histogram from the distance between the agents during the course of the behavior, over all trials. From this, the probability and the entropy were computed in the same way as neural entropy and normalized by the maximum entropy of log(100). While the main manuscript does not use this measure, see supplementary results below for analysis of relationship between behavioral complexity and interaction.

1.5 Analysis with "ghost" partner

In order to delineate the role played by interdependent interaction on internal complexity as opposed to independent interaction, agents that showed high levels of internal complexity in the presence of a partner were tested under a "ghost" condition. In this case, from the genotype of the best solution of a particular run, only one agent was constructed. This agent was placed in an environment in the presence of another agent. However, the other agent, not being constructed from the genotype, was not an active system that was behaving in this environment. Instead, that agent was replaying pre-recorded behavior from the trials that were conducted after optimization. This is referred to the "ghost" agent. In order for the active agent, whose entropy is being measured, to also not repeat its behavior from those trials, the two agents were started at different random initial angles from each other, while keeping the initial distance the same. Same as the evolutionary fitness evaluation, 4 trials were conducted, and the neural entropy of the active agent was measured based on its behavior in the presence of a "ghost". This setup allows neural entropy to be measured under similar conditions of sensory complexity that the agents experience during interdependent interaction, while at the same time removing any possibility for the same.

1.6 Curve fitting and statistical testing

IBM SPSS Statistics software was used for regression modeling and statistical testing. The distribution of evolved entropies between the different setting of isolation and interaction were statistically tested by comparing means using a two-tailed t-test with significance declared if $p \leq 0.05$. For the regression model, coefficients were fit based on 95% confidence bounds and an analysis of variance (ANOVA) over the regressors, and the residuals was conducted and tested using an F-statistic test to check against all

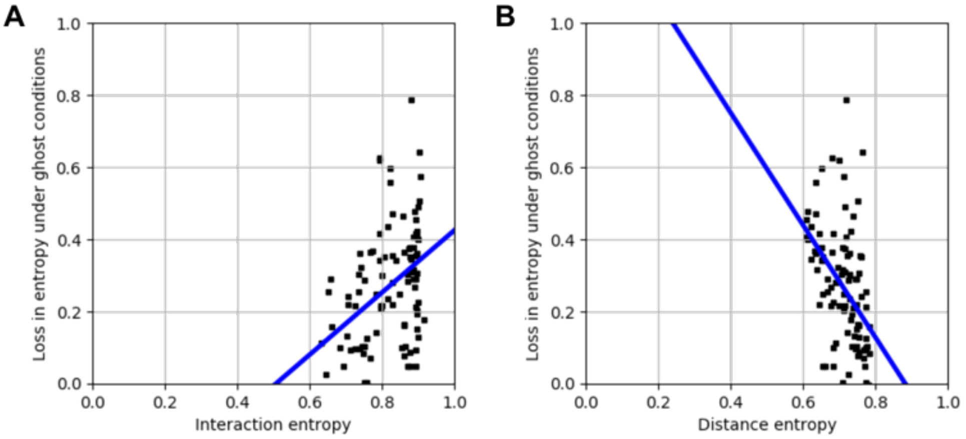

Figure S1. Relationship between interdependent interactions, behavioral complexity and internal complexity. [A] A positive correlation between interaction entropy and loss in entropy under ghost conditions suggests that interdependence increases with internal complexity. [B] A negative correlation between distance entropy (behavioral complexity) and loss in entropy under ghost conditions suggests that interdependence is hindered by greater behavioral complexity. Thus, interdependent interaction is achieved by trading-off internal complexity for behavioral complexity.

coefficients being 0. Further, each co-efficient was individually tested against being 0, using a two-tailed t-test with significance declared for p-values below 0.05.

2 SUPPLEMENTARY RESULTS

2.1 Interdependent interaction occurs at a balance between behavioral complexity and internal interaction complexity

Greater loss in entropy between the active and the ghost conditions corresponds to greater levels of interdependent interaction. In order to study the relationship between an agent's complexity during interaction and the level of interdependence in it, we performed a linear fit between the two (Fig. S1A). A linear regression analysis showed that interaction entropy significantly predicted loss in entropy under the ghost condition (R2=0.16, F(1,98), p¡.01) and beside the constant, interaction entropy was specifically tested using a t-statistic showing that it significantly predicted loss in entropy under ghost conditions (two-tailed t-test $p \ll 0.05$, see supplementary tables for detailed statistical test results). This suggests that an increased level of internal complexity typically corresponds to greater levels of interdependent interaction.

On the other hand, when the entropy in distance between the agents, which is a proxy for behavioral complexity, was fit against loss in interaction entropy under ghost conditions, a negative correlation was revealed (Fig. S1B). The linear fit was found to be statistically significant (R2=0.208, F(1,98), $p \leq .01$) and distance entropy independently tested showed significant predictability of loss in entropy under ghost conditions (two-tailed t-test, $p \ll 0.05$, see supplementary materials for detailed statistical test results). Thus, it can be said that greater levels of complexity in behavior hinders interdependent interaction. From the two trends we've shown in the factors that predict interdependent interaction, we posit that while mutually interdependent interaction enhances internal complexity, it is constrained by behavioral complexity that hinders the same. In other words, productive social behavior occurs at a fine balance between behavioral and internal complexity.

3 SUPPLEMENTARY TABLES AND FIGURES

3.1 Figures

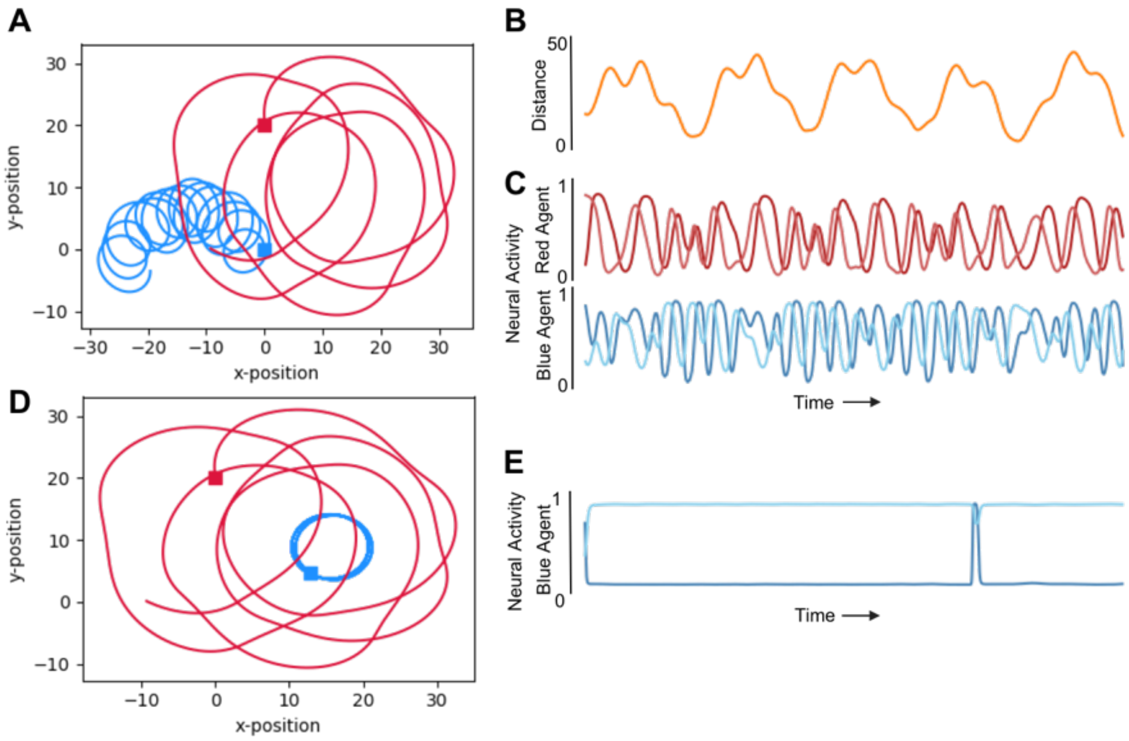

Figure S2. Illustration of an agent's behavior, neural activity and distance traces in interaction and its performance and neural activity in the ghost condition. [A] Behavior in this agent involved both agents performing coordinated spiraling movements such that their they maintained their relative distance within a particular range as shown in [B]. [C]The neural activity of the two agents shows chaotic dynamics that is, in principle, not possible for two dimensional dynamical systems on their own. [D] When the red agent plays back its behavior from the trail show in A, and they start from a different initial relative position, behavior in the blue agent is significantly compromised. [E] Neural activity in the ghost condition shown very little dynamics showing that the system settled into a fixed-point attractor. This particular agent had an interaction entropy of 0.8790 for its dynamics in C and a ghost entropy = 0.0901

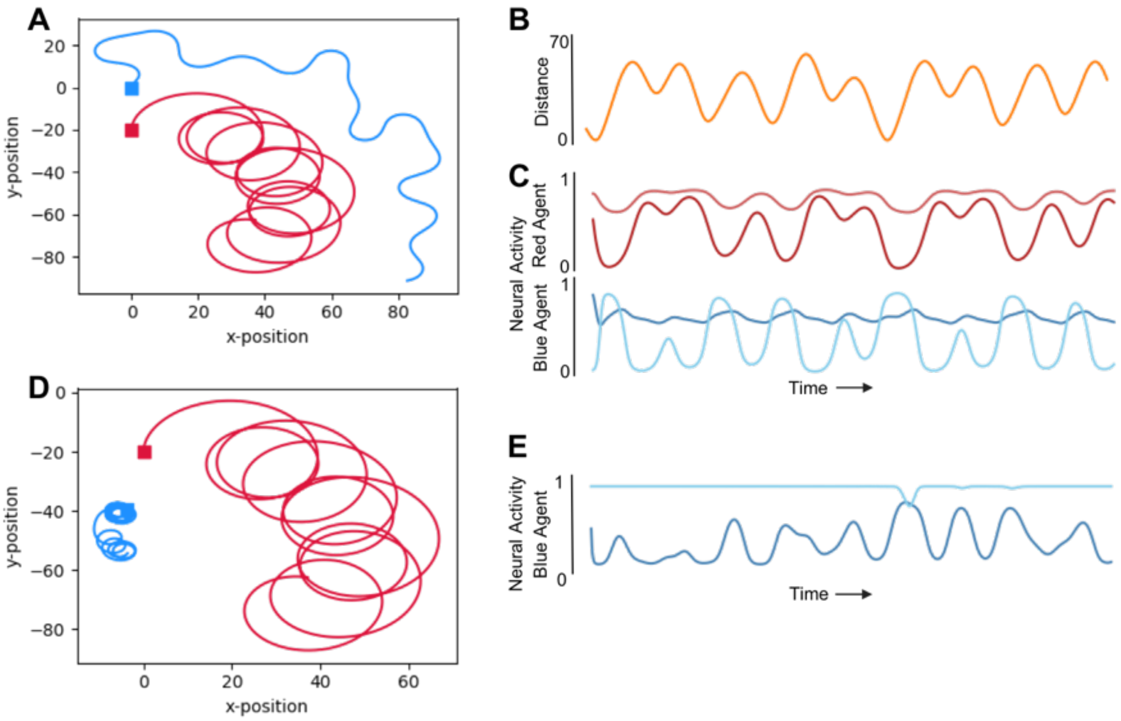

Figure S3. Illustration of an agent's behavior, neural activity and distance traces in interaction and its performance and neural activity in the ghost condition. [A] the red agent has a spiral behavioral trajectory whereas the blue agent doesn't. However, they move in a such a pattern with relation to each other as if they are moving towards something together maintaining the distance between them in a particular range. [B] The distance between these agents over time shows that they are moving back and forth towards and away from each other while their overall behavior trajectory shows they are moving forward in effect. [C] Neural activities of both read and blue agents shows chaotic dynamics in this case too. [D] Under ghost conditions, the red agent is repeating its behavior from trial shown in A. Unlike figure S1, the blue agent relies only partly on the red for behavioral as well as neural complexity. This is also reflected in its neural activity shown in E. This agent had an interaction entropy of 0.8281 and a relatively high entropy in ghost condition of 0.3560.

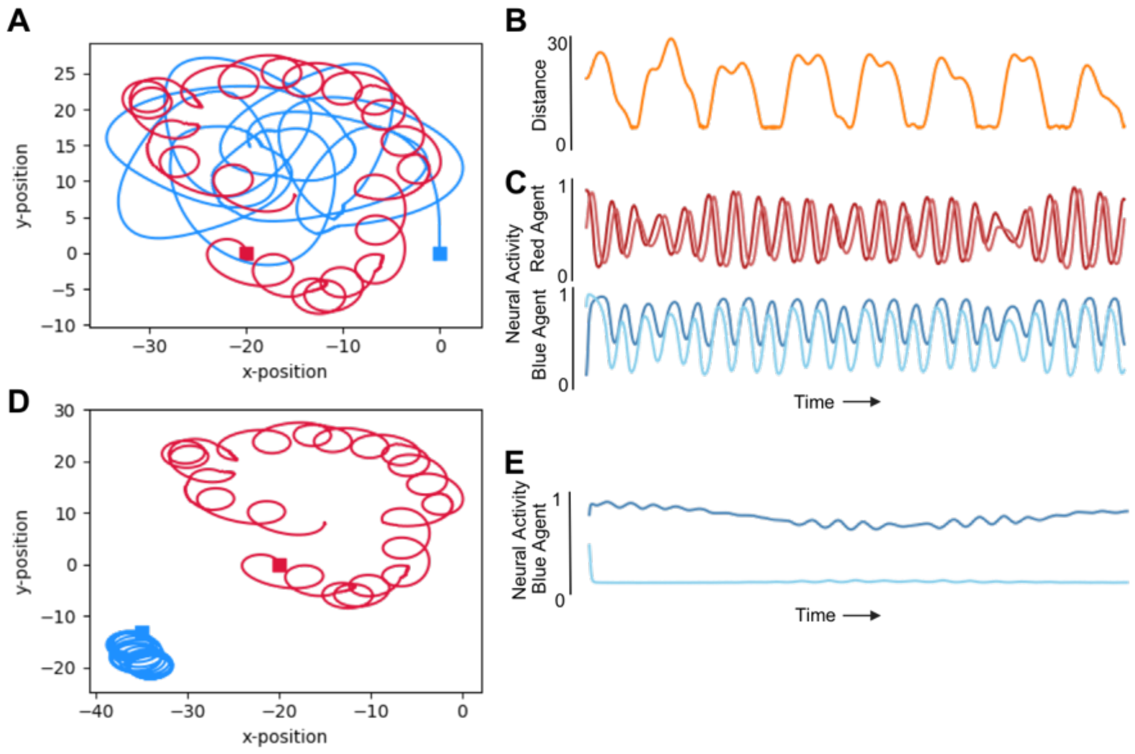

Figure S4. Another illustration of an evolved agent in interaction and under the ghost condition. [A] Both agents show rather complex behavioral complexity involving several spirals and collisions. [B] the complex behavioral traces, however, can be seen manifesting as the familiar periodic pattern of moving towards and away from each other. [C] The complexity in the neural activity in this case is less that shown in figures S1 and S2 with an interaction entropy of 0.7249. [D] Under the condition of facing a ghost partner, the blue agent seems to still cope and maintaining a rather high internal complexity (neural activity shown in E) suggesting the agent did not rely on the partner as much for increasing its internal complexity. The ghost entropy from all 4 trials (only 1 shown here) for this agent was 0.508.

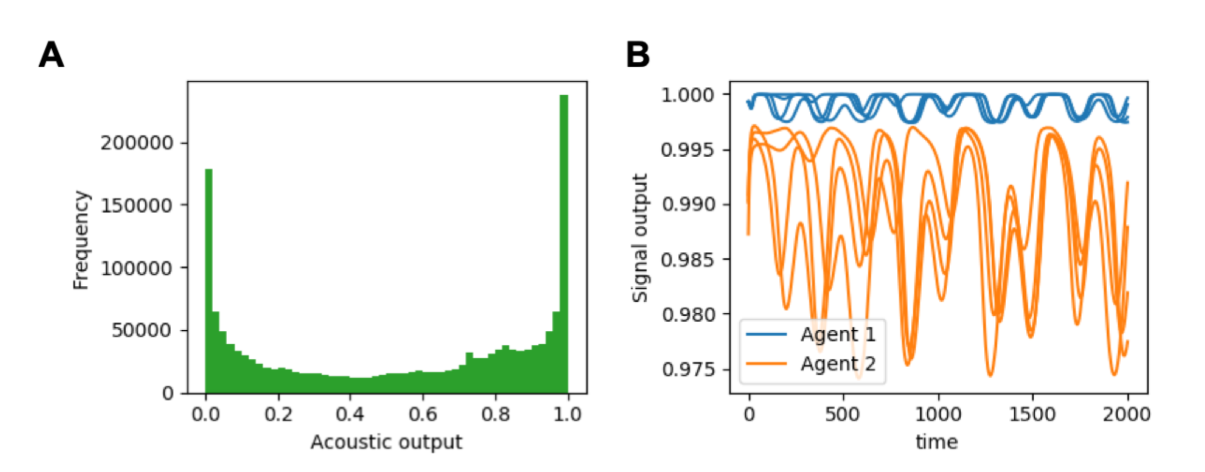

Figure S5. Emergent interaction upon evolving for neural complexity in the presence of interaction. [A] Distribution of strength of acoustic signal emitted by all 200 agents (one pair each of 100 different runs) that were evolved with interaction. While the acoustic signal show maximum frequency on either saturated ends, it can be seen that the acoustic signal bears a non-saturated value for a significant amount of time. [B] Illustration of acoustic signal for pair of agents shown in Fig. S3. Each trace corresponds to the acoustic signal emitted during one trial of the simulation by each agent in the presence of the other.

3.2 Tables

3.2.1 Statistical test results comparing distributions of neural entropies when (a) agents are evolved in isolation, (b) agents are evolved in interaction and (c) interactive agents evaluated in isolation

	Levene's test for Equality of Variances		t-test for equality of means						
	F	Sig	t	df	Sig. 2-tailed	mean difference	std. error difference	95% confidence interval	
								lower	upper
Equal variances assumed	195.013	.00	52.23	198	.000	.4233	.008	.407	.439
Equal variances not assumed			52.23	106	.000	.4233	.008	.4071	.4392

Table S1. Independent samples test between agents evolved in isolation and agents evolved in the presence of another agent

	Levene's test for Equality of Variances		t-test for equality of means						
	F	Sig	t	df	Sig. 2-tailed	mean difference	std. error difference	95% confidence interval	
								lower	upper
Equal variances assumed	176.537	.00	-26.2	198	.000	-0.3085	0.0117	-0.3317	-0.2853
Equal variances not assumed			-26.2	102.26	.000	-0.3085	0.0117	-0.3319	-0.2852

Table S2. Independent samples test between agents evolved in isolation and interactive agents evolved whose neural entropy is measured in isolation.

3.2.2 Statistical test of linear regression fit - Interaction entropy versus loss in entropy under ghost condition

R	R^2	Adjusted R^2	Std. error of estimate	Change Statistics				
				R^2 change	F change	df1	df2	Sig. F change
.411	.169	.160	.1529	.169	19.913	1	98	.000

Table S3. Model Summary for linear fit between interaction entropy and Loss in entropy under ghost condition. Predictors: (Constant), Interaction Entropy

Model	Sum of squares	df	Mean square	F	Sig.
Regresion	.466	1	.466	19.913	.000
Residual	2.294	98	0.023		
Total	2.760	99			

Table S4. ANOVA on variance about dependent variable “Loss in entropy under ghost condition” explained by regressors and residuals

	Unstandardized Coefficients		Standardized Coefficients	t	Sig.	95% confidence interval for B	
	B	Std.error	Beta			Lower bound	Upper bound
(Constant)	-0.437	0.159		-2.753	.007	-0.751	-0.122
Interaction Entropy	0.862	0.193	0.411	4.462	.000	0.479	1.245

Table S5. Coefficents from the linear model fit with statistical test for the predictability of Loss in entropy under ghost conditions

3.2.3 statistical test of linear regression fit - Distance entropy versus loss in entropy under ghost condition

R	R^2	Adjusted R^2	Std. error of estimate	Change Statistics				
				R^2 change	F change	df1	df2	Sig. F change
.456	.208	.200	.1493	.208	25.683	1	98	.000

Table S6. Model Summary for linear fit between distance entropy and Loss in entropy under ghost condition. Predictors: (Constant), Interaction Entropy

Model	Sum of squares	df	Mean square	F	Sig.
Regresion	.573	1	.573	25.683	.000
Residual	2.187	98	0.022		
Total	2.760	99			

Table S7. ANOVA on variance about dependent variable "Loss in entropy under ghost condition" explained by regressors and residuals.

	Unstandardized Coefficients		Standardized Coefficients	t	Sig.	95% confidence interval for B	
	B	Std.error	Beta			Lower bound	Upper bound
(Constant)	1.374	0.219		6.279	.000	0.940	1.808
Distance Entropy	-1.556	0.307	-0.456	-5.068	.000	-2.166	-0.947

Table S8. Coefficents from the linear model fit with statistical test for the predictability of Loss in entropy under ghost conditions

REFERENCES

Di Paolo, E. A. (2000). Behavioral coordination, structural congruence and entrainment in a simulation of acoustically coupled agents. *Adaptive Behavior* 8, 27–48
